# Benign epithelial oral lesions – association with human papillomavirus

**DOI:** 10.4317/medoral.22817

**Published:** 2019-04-24

**Authors:** Alicia-Rumayor Piña, Felipe-Paiva Fonseca, Flávia-Sirotheau-Corrêa Pontes, Hélder-Antônio-Rebelo Pontes, Fábio-Ramôa Pires, Adalberto-Mosqueda Taylor, José-Manuel Aguirre-Urizar, Oslei-Paes de Almeida

**Affiliations:** 1 Oral Pathology Section, Department of Oral Diagnosis, Piracicaba Dental School, University of Campinas. Brazil; 2 Department of Oral Surgery and Pathology, School of Dentistry, Universidade Federal de Minas Gerais. Brazil; 3 Service of Oral Pathology, João de Barros Barreto University Hospital, Federal University of Pará. Brazil; 4 Oral Pathology, Department of Surgery and Diagnosis, Dental School, State University of Rio de Janeiro. Brazil; 5 Departamento de Atención a la Salud, Universidad Autónoma Metropolitana Xochimilco, México; 6 Departamento de Estomatología II. Facultad de Medicina y Enfermería. Universidad del País Vasco/EHU. España

## Abstract

**Background:**

The presence of human papilloma virus in benign oral lesions has been studied by different techniques obtaining extremely variable results. The objective of this study was to determine the presence of human papillomavirus in 83 cases of benign hyperplastic epithelial oral lesions.

**Material and Methods:**

Eighty-three oral lesions with clinical or histopathological features suggestive of HPV infection were retrieved from the files of four oral pathology services. Demographic data were obtained from patient´s medical charts. All cases had available clinical image, H&E preparations and paraffin blocks with enough tissue for HPV detection by in situ hybridization, and immunohistochemical reactions for Ki67.

**Results:**

Episomal positivity for wide spectrum HPV was observed in 24% of the cases; most of them (70%) HPV 6/11 positive. HPV 16/18 was not detected. Condyloma acuminatum was the most common lesion associated with HPV (75%), followed by verruca vulgaris (15%), squamous papilloma and multifocal epithelial hyperplasia, 5% each. Koilocytes were identified in all the HPV positive cases. Ki67 showed an abnormal proliferation pattern in 90% of the HPV positive cases; most of them (70%) showing groups of proliferating cells in focal superficial regions, and in 20% positivity was seen almost in the whole thickness of the epithelium. HPV negative cases showed Ki67 positive cells restricted to the basal layer.

**Conclusions:**

Regarding oral lesions associated with HPV, condyloma is the most common lesion expressing low-risk subtypes. The etiology of squamous papilloma remains controversial as HPV was found in 1.9% of the cases. The identification of koilocytes and the pattern of expression of Ki67 reflect HPV infection and are helpful for classification. Papillary oral lesions not associated to HPV deserve further studies to better clarify its etiology.

** Key words:**Human papillomavirus, condyloma, papilloma.

## Introduction

The World Health Organization (WHO) recognizes four human papillomavirus (HPV) related oral lesions; squamous cell papilloma (SP), condyloma acuminatum (CA), verruca vulgaris (VV) and multifocal epithelial hyperplasia (MFEH). These lesions are benign hyperplastic exophytic proliferations of the oral epithelium associated with different subtypes of HPV. Papilloma tend to be solitary and pedunculated, the percentage of HPV positive lesions is highly variable, ranging from 0% to 100%, with an average of 34% depending on the detection technique. Low-risk HPV 6 and 11 are the most commonly found subtypes. Histologically SP show sharp-pointed digitiform projections of the epithelium without evident koilocytes or keratohyaline granules. VV is uncommon intraorally, it affects mainly lip vermillion, clinically, a white rough surface is seen, often presenting as multiple or clustered lesions. Features that help distinguishing VV from SP are a thick layer of orthokeratin, prominent keratohyaline granules and presence of koilocytes. VV is associated with HPV 2, 4, 40 and 57. Oral CA corresponds to the counterpart of genital condyloma, usually HPV 6/11 positive, and it can be differentiated from SP by its bulbous, rounded and short projections (1-4). MFEH has well established features and it is associated with HPV 13 and 32, it affects specific groups of individuals in a few regions of the world, particularly Eskimos and Amerindians of North, South and Central America, 5% of the cases may show papillary/verrucous surface, but not predominantly, as smooth flat papules tend to outnumber the papillary lesions. Prominent acanthosis is a key feature as well as the so called mitosoid figures, which are considered highly characteristic of this condition (5-7). Clinical and histopathological features are supposed to be enough to differentiate these groups of oral epithelial lesions; however, in an oral pathology daily work this differentiation can be difficult, and misuse of terms makes this subject confuse. We herein describe and discuss the association of HPV with 83 benign epithelial hyperplastic oral lesions to better clarify this group of oral mucosal lesions.

## Material and Methods

This was a retrospective study based on eighty-three cases of oral lesions with clinical and/or histopathological features suggestive of HPV association. The cases were retrieved from the files of four oral pathology services.  Table 1 summarizes the clinical features of the cases. In addition, for histological comparison, 13 cases of genital condyloma acuminatum from penis (5), anus (5), and vulva (3) previously known to be HPV 6/11 positive were included. All the oral cases had available clinical image, H&E preparation and paraffin blocks with enough tissue for HPV detection by in situ hybridization (ISH) and for immunohistochemical reactions. The cases were classified according the criteria defined by the WHO, considering squamous papilloma, condyloma acuminatum, verruca vulgaris, and multifocal epithelial hyperplasia.

**Table 1:**
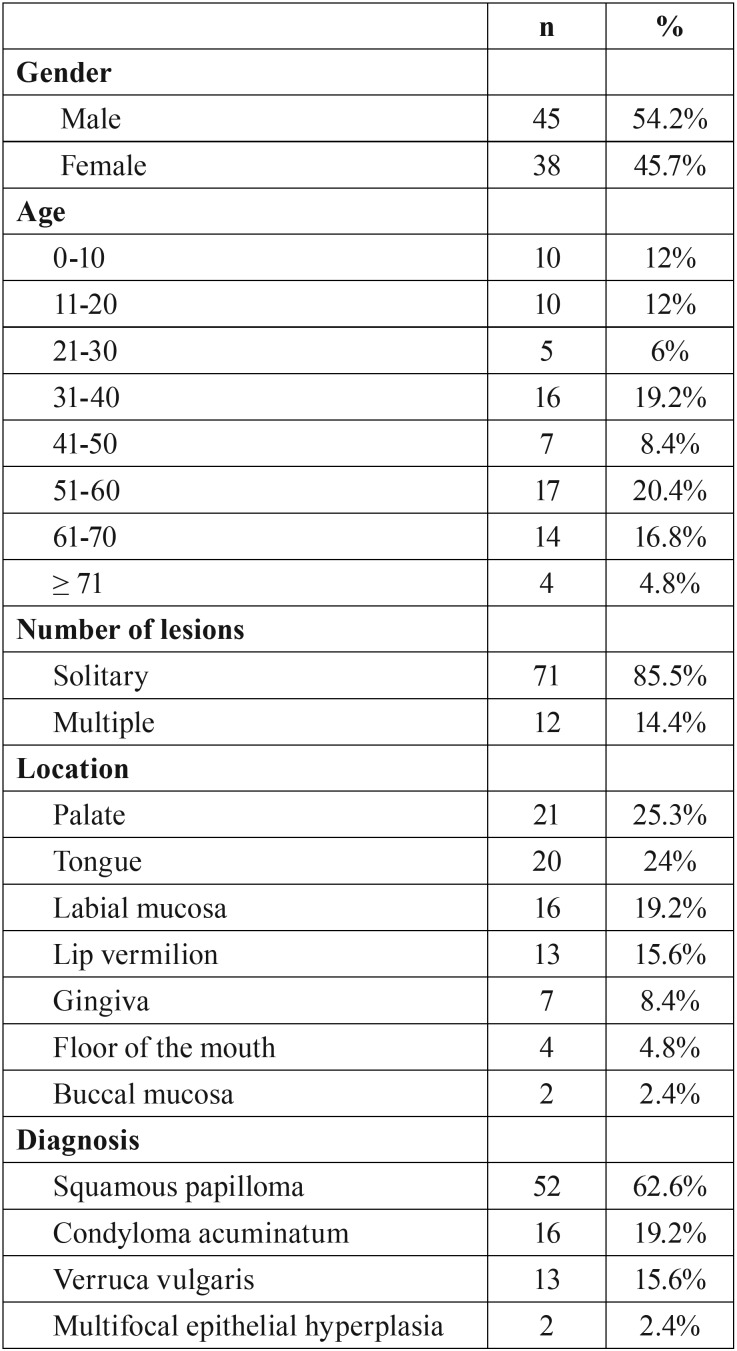
Clinical features.

In situ hybridization was performed in 5µm sections using a wide spectrum (WS) probe that includes genotypes 6, 11, 16, 18, 31, 33, 35, 39, 45, 51 and 52, and two specific probes; HPV 6/11 and HPV 16/18 (Y1404, Y1411, Y1412, Dako, Carpinteria, CA). The Catalyzed Signal Amplification System (K0620; Dako, Carpinteria, CA) was used for visualization. All procedures were performed following the manufacturer´s protocol. Cases of uterine cervix and tongue squamous cell carcinoma were used as positive and negative controls, respectively. The presence of a complete nuclear (episomal) or punctate/dots (integrated) dark brown signals within the epithelial cells were considered positive (8). Immunohistochemistry (IHC) for Ki67 (Dako, MIB-1, 1:100) was also performed in 3µm sections following a known protocol (9).

## Results

There were 45 males and 38 females with a mean age of 41 years (range 2-78 years). Solitary lesions prevailed, and palate, tongue and lip vermilion were the most affected sites. Twenty (24%) from 83 cases of hyperplastic epithelial oral lesions were positive for the wide spectrum probe, and 14 (70%) out of these were of the 6/11 subtype. In all the cases positive cells had an episomal pattern. The high-risk specific probe 16/18 was not positive in any case.  Table 2 summarizes the features of the HPV associated oral lesions.

**Table 2:**
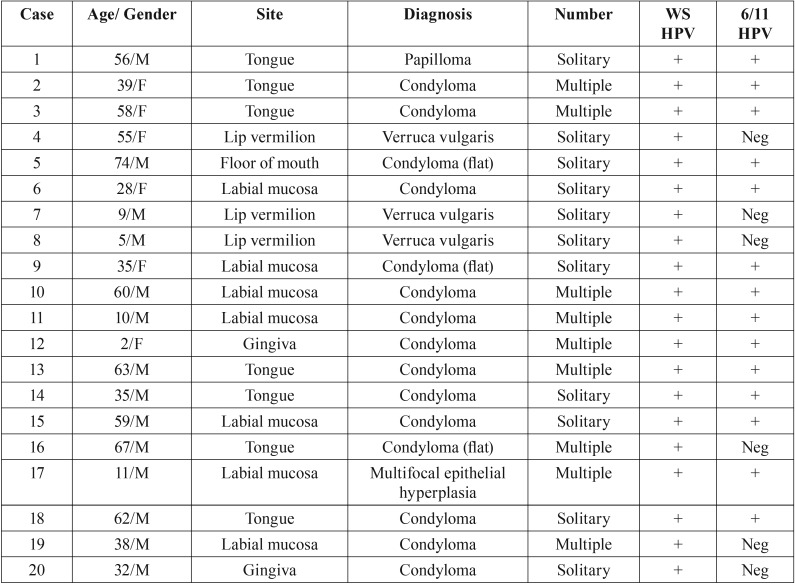
Features of the HPV positive cases.

**Figure 1:**
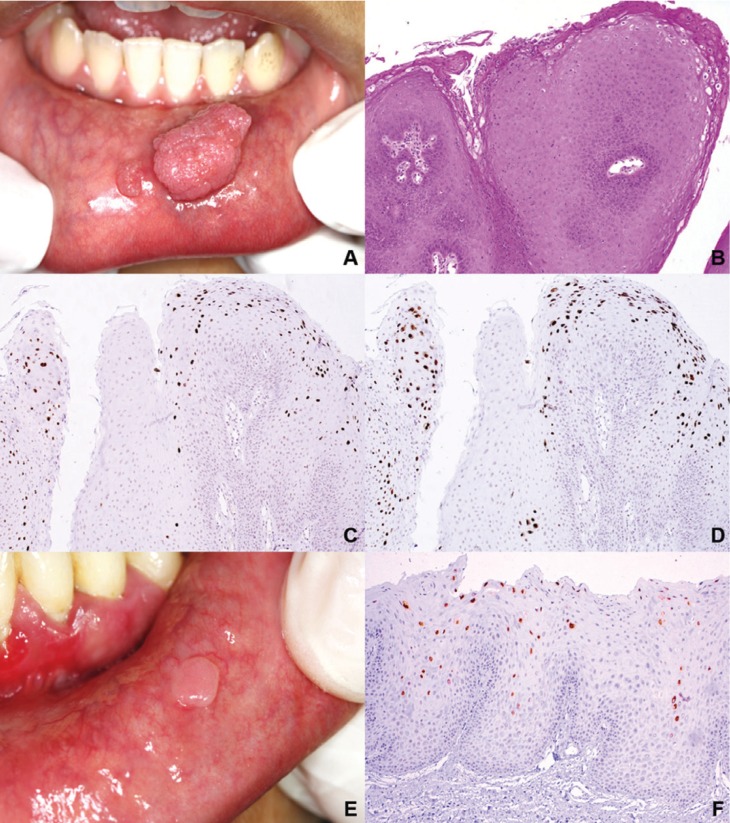
Condyloma acuminatum (case 11). A. Two nodular lesions with slightly papillary surface. B. Blunt hyperplastic epithelium with papillary rounded projections showing superficial koilocytes (H&E, 50x) C. Positivity for wide-spectrum HPV (ISH, 100x). D. Positivity for HPV 6/11 (ISH, 100x). E. Flat condyloma (case 9). Solitary smooth surfaced nodule. F. Flat acanthosis with blunt processes showing positivity for HPV 6/11 (ISH, 100x).

**Figure 2:**
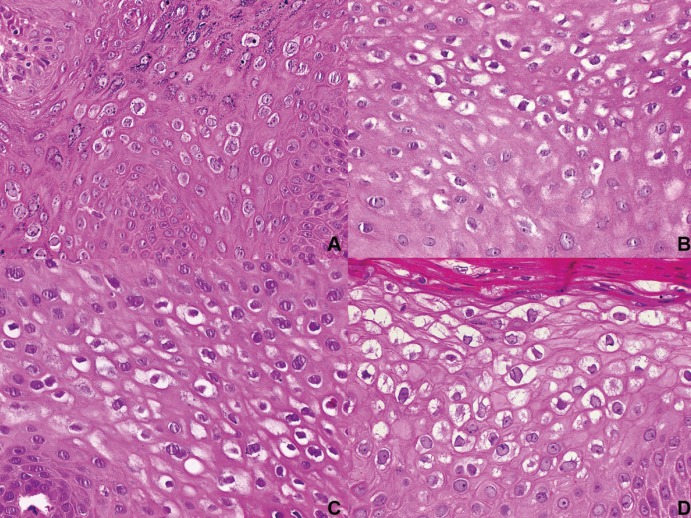
Koilocytes in oral and genital lesions. A. Labial VV. Coarse keratohyaline granules are evident (H&E, 400x). B. Condyloma of ventral tongue (Case 13, H&E, 400x). C. Flat condyloma of floor of the mouth (Case 5, H&E, 400x). D. Penile condyloma (H&E, 400x).

Regarding to diagnosis, cases classified as condyloma were the most associated with HPV, 75% were positive for the 6/11 specific probe (Fig. 1A-F). Three cases (23%) of verruca vulgaris were positive only for the wide spectrum probe, while one case each of SP and MFEH were positive for both probes. Koilocytes were more commonly observed in condylomas and MFEH, followed by VV, most of them in the upper portion of the spinous stratum (Fig. 2).

**Figure 3:**
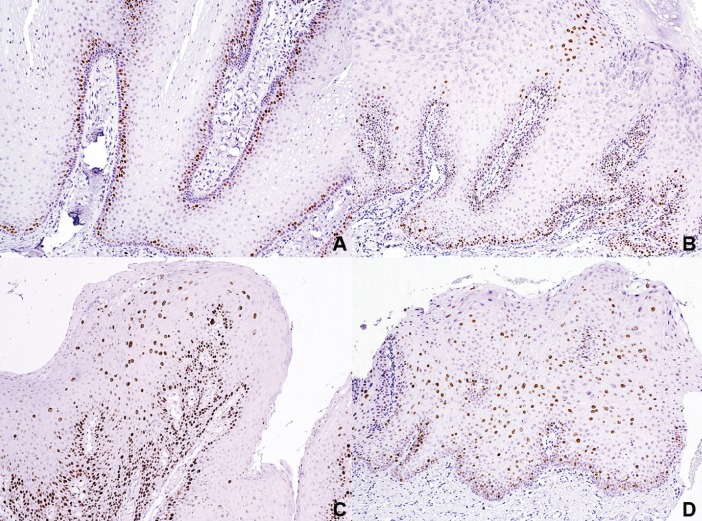
Immunohistochemical expression patterns of Ki67. A. HPV negative SP showing proliferating cells restricted to the basal layer (IHC, 100x). B. Labial VV showing basal Ki67 and a focal group of cells near the surface (IHC, 100x). C. CA showing proliferating cells in the basal and spinous layer (Case 11, IHC, 100x). D. HPV positive flat condyloma showing acanthosis containing proliferating cells in the whole thickness of the epithelium (case 5) (IHC, 100x).

Ki67 highlighted proliferating cells restricted to the basal layer in 67.4% of the cases, mainly corresponded to SP. In 27 cases (32.5%) a more diffuse pattern was observed, in 22 cases besides the basal layer, focal groups of superficial cells expressed Ki67 corresponding to condyloma and VV, while in 5 cases positivity was observed in the whole thickness of the epithelium (Fig. 3). The latter corresponded to CA, 3 of them showing flat acanthosis instead a papillary morphology. From the cases with an abnormal pattern of expression of Ki67, 66.6% were associated with HPV.  Table 3 summarizes the findings by diagnosis. Genital CA also showed Ki67 positive cells besides the basal layer; in 54% of the cases the expression was observed in the whole thickness of the epithelium, while in 38% in focal superficial areas.

**Table 3:**
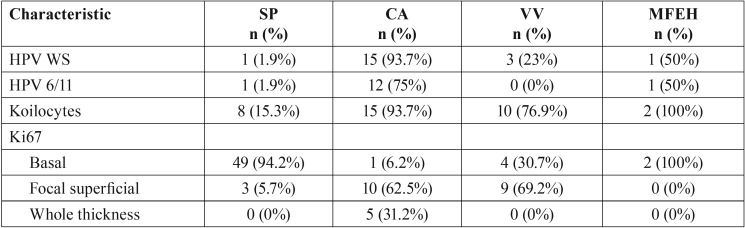
Features according diagnosis.

## Discussion

Most oral papillary lesions share low-risk HPV subtypes (6/11), this raises the question whether these lesions belong to the same spectrum with different clinical presentation (3). We found HPV in 24% of 83 cases or benign hyperplastic epithelial oral lesions, similar to data reported in other studies about oral warts. Low risk subtypes were seen in 70% of the wide spectrum positive lesions, mostly cases of condyloma acuminatum. Regarding to squamous papilloma, positivity for HPV is extremely variable in the literature (10-12), we found this association only in one case. Reported prevalence of high-risk HPV in oral samples are controversial, these subtypes are most commonly found in cervical and anogenital malignancies. In our study, they were not detected in any case, thus, indicating a possible mechanism of natural resistance of the oral epithelial cells to these aggressive subtypes. This is a possible explanation for the low association of oral cancer and HPV, different from the cervix and oropharynx (13-15).

Cases positive for the wide-spectrum probe and negative for the specific probes, half of them with diagnosis of VV were in some way expected because these probes did not include the genotypes typically associated with this lesion: 2 and 57 (16). These cases may be probably positive for other subtypes included in the wide spectrum probe, such as 31, 33, 35, 39, 45, 51 or 52¸ nevertheless these types are recognized as carcinogenic. None of the VV showed positivity for HPV 6/11, however, these types have been identified by ISH in 15% of oral VV as reported previously (10). Regarding the 2 cases of MFEH, we expected negativity for all the probes used, because these lesions are known to be associated with HPV 13/32 (7), probes that are not commercially available. Nevertheless, one case affecting an 11-year-old boy was positive for the wide spectrum and 6/11 probes. As co-expression of genotypes has been observed, we could not rule out the presence of types 13 and 32, as we did not investigate with this probe.

Abundant koilocytes and an abnormal expression of Ki67 are features identified in most of the HPV associated lesions. In CA these viral altered cells were seen in the upper layers of epithelial crevices, as reported previously. Koilocytes have been identified in approximately 87% of oral condyloma and in a range from 27% to 45% of oral papilloma (12,17). We found these cells in 93.7% of oral condyloma; and in 15.3% of squamous papilloma. In genital warts koilocytes are usually abundant and evident, while in oral mucosa these cells are more difficult to identify with certainty, because they can be confused with vacuolated epithelial cells, which are common in the oral epithelium. However, strict criteria for koilocyte identification should be applied; perinuclear clear halo, nuclear enlargement, hyperchromasia, irregular nuclear outlines, and multilobation should be identified (18).

Ki67 showed to be interesting to indicate association with HPV, as almost 70% of the cases showing a non-restricted basal layer positivity, resulted to be HPV associated. It is important to consider that these lesions should be distinguished from epithelial dysplasias not associated with HPV.

One final point that deserves further consideration is that the squamous papillomas were mostly HPV negative. This is probably associated to the fact that from the more than 170 genotypes described, we used in this study only a limited number of probes, and it is unknown with certainty what percentage of the genome is common for those genotypes not commercially available. High discrepancies are found when using methods such as ISH or PCR, as the latter possess a higher sensitivity but lower specificity, and studies using PCR shows higher percentages of positivity (19). In conclusion, low-risk HPV 6/11 are the most common subtypes found in the oral cavity, even though, a small percentage of hyperplastic papillary lesions are associated with HPV, they comprise a wide group of lesions that deserves further studies to better clarify its classification and etiology.
